# Selective and Efficient Neural Coding of Communication Signals Depends on Early Acoustic and Social Environment

**DOI:** 10.1371/journal.pone.0061417

**Published:** 2013-04-22

**Authors:** Noopur Amin, Michael Gastpar, Frédéric E. Theunissen

**Affiliations:** 1 Helen Wills Neuroscience Institute, University of California, Berkeley, California, United States of America; 2 Department of Electrical Engineering and Computer Science, University of California, Berkeley, California, United States of America; 3 Psychology Department, University of California, Berkeley, California, United States of America; Australian National University, Australia

## Abstract

Previous research has shown that postnatal exposure to simple, synthetic sounds can affect the sound representation in the auditory cortex as reflected by changes in the tonotopic map or other relatively simple tuning properties, such as AM tuning. However, their functional implications for neural processing in the generation of ethologically-based perception remain unexplored. Here we examined the effects of noise-rearing and social isolation on the neural processing of communication sounds such as species-specific song, in the primary auditory cortex analog of adult zebra finches. Our electrophysiological recordings reveal that neural tuning to simple frequency-based synthetic sounds is initially established in all the laminae independent of patterned acoustic experience; however, we provide the first evidence that early exposure to patterned sound statistics, such as those found in native sounds, is required for the subsequent emergence of neural selectivity for complex vocalizations and for shaping neural spiking precision in superficial and deep cortical laminae, and for creating efficient neural representations of song and a less redundant ensemble code in all the laminae. Our study also provides the first causal evidence for ‘sparse coding’, such that when the statistics of the stimuli were changed during rearing, as in noise-rearing, that the sparse or optimal representation for species-specific vocalizations disappeared. Taken together, these results imply that a layer-specific differential development of the auditory cortex requires patterned acoustic input, and a specialized and robust sensory representation of complex communication sounds in the auditory cortex requires a rich acoustic and social environment.

## Introduction

Recent studies have shown that low-level [1], [2], [3], [4] and high-level auditory systems [5], [6], [7] are tuned to natural sounds, such that single or ensembles of neurons respond optimally to sound features pertaining to an animal's natural and behaviorally-relevant acoustic environment. However, it still remains unclear to what degree the brain adapts to the environment experienced by the animal during its development and to what degree it is hardwired for more universal natural statistics. We were interested in addressing the role of development, and specifically the role of early patterned sensory input, in establishing neural tuning for behaviorally-relevant communication signals found in the auditory system of adult animals [8], [9], [10]. Although recent studies in the rodent auditory cortex have made considerable progress in revealing environmental influence on the development of simple frequency topographic maps or simple temporal tuning properties [11], [12], [13], [14], [15], [16], [17], their functional implications for neural processing in the generation of ethologically-based perception and behavior remain unexplored.

We chose to study the emergence of neural tuning for communication signals in the songbird model. Given that young songbirds use inherent auditory preferences for conspecific songs to guide the learning of complex acoustic and vocal song communication from conspecific adults [18], [19], songbirds are ideal for studying the innate and learned components of neuronal mechanisms involved in the comprehension and production of complex vocalizations (and reminiscent of human speech learning [20]). Moreover, neurons in the auditory system of adult zebra finches show varying degrees of specialization for processing species-specific vocalizations [21], and neural representation for behaviorally-relevant sounds has been shown to be plastic during development and adult learning [22], [23], [24], [25]. In our previous work, we also showed that adult auditory forebrain neurons respond more robustly to song over synthetic stimuli designed to match lower-order song statistics [26], and that this selectivity emerges during development [27]. Furthermore, single neurons transmit more mutual information about song and song-like sounds than about broadband noise [28].

**Figure 1:**
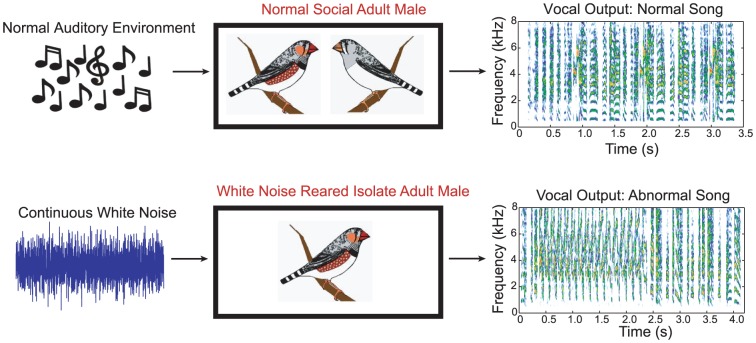
Experimental setup included wn-rearing and social isolation. Experimental setup included normal acoustic and social rearing for control birds, and a continuous white-noise exposure and social isolation for wn-reared birds. The song output of one control male and one wn-reared male are shown in spectrogram form on the right: the control males sang a normal song, typical of normal/tutored zebra finches; and the wn-reared males sang a scratchy, perseverative song, typical of untutored/isolate song. Although we did not systematically study the differences in vocal output, we used song production as a means to verify the effectiveness of the white noise exposure and isolation since song development in males depends on both natural auditory spectro-temporal cues and social interactions.

In this study, we raised zebra finches in isolation and in continuous unstructured white noise until adulthood (‘wn-reared’; Figure 1) and then recorded neural responses in the field L complex (primary auditory cortex analog) to conspecific song and statistically-matched synthetic sounds. We compared neural selectivity, spectro-temporal receptive fields (STRFs), and information/redundancy measures obtained from birds raised in noisy environments to those obtained from normal, social adults (‘controls’). We provide the first evidence that the absence of patterned auditory stimulation during postnatal life did not play a role in establishing neural tuning to simple frequency-based synthetic sounds in all the laminae or spectro-temporal tuning in the thalamorecipient lamina, but dramatically reduced the neural selectivity for natural sounds, such as song, over some synthetic sounds in the more downstream laminae of field L. Moreover, we provide the first demonstration that the efficient and sparse neural representation for species-specific vocalizations found in control animals depended on exposure to these native sounds.

## Materials and Methods

### Animal Procedures

All animal procedures were approved by the Animal Care and Use Committee at UC Berkeley (Protocol Number: R241-0113C). This study was carried out in strict accordance with the recommendations in the Guide for the Care and Use of Laboratory Animals of the National Institutes of Health. White noise (20 Hz to 10 kHz) was generated via a white noise generator (Random Noise Generator Model ST-NG1 from Radio Design Labs) and streamed continuously through a bookshelf speaker (JBL Northridge 2-way speakers) in each of the acoustic isolation boxes (dimensions: 29.5“x24”x19.5”, from Acoustic Systems) in which we housed the subjects. Recording samples of the acoustic environment showed that the power spectra of the noise were relatively flat in power (±5 dB) up through 10,000 Hz (the upper limit of zebra finch hearing ability), thereby ensuring a non-structured, unnatural acoustic rearing environment for the songbirds.

The white noise was played at sound levels of 80–85 dB SPL. These moderate sound levels corresponded to the upper range of noise levels found in urban environments (www.noise.org) and are sufficient to dramatically mask incidental environmental sounds produced by the animal's movements (e.g. hopping sounds) as well as its own vocalizations. When choosing these sound levels for the noise, we realized that the masking of vocalizations would be substantial but still only partial (SNR ∼1) and that zebra finches, like other vertebrates, can increase amplitude levels of vocalization in response to increased levels of noise [29]. Our goal however was not to eliminate all natural sound input but to drastically and systematically affect the exposure to the natural spectral-temporal structure. Finally and importantly, these sound levels were well below levels that would damage hair cells [30]. Behavioral studies in the birdsong field have also shown that exposing adult zebra finches to chronic loud white noise does not change their song drastically [31] and the memory of the tutor syllables survives such auditory perturbations [32].

Fourteen birds (7 males and 7 females) were born in these noisy environments and raised by the genetic mother in the company of the rest of the brood (and sometimes with another adult female helper bird) until they fledged (about 18–21 days). One to 3 days after fledgling (also about the time of weaning), the young bird was isolated and raised in his/her own acoustic isolation booth with continuous, streaming white noise through adulthood (condition termed ‘wn-reared’) (see Figure 1). Our goal with the social isolation was similar to our noise exposure goal, in that we aimed to drastically reduce (but not completely eliminate) social interactions for the majority of the zebra finches' development, with complete isolation occurring from weaning to adulthood. Ten birds (5 males and 5 females) were wn-reared until 4 months of age, and 4 others (2 males and 2 females) until 6 months of age, after which they were used in acute neurophysiological recordings. We also recorded the “isolate” song for a majority of the male subjects (n = 6), and examined the impact of wn-rearing on vocal song output. To record the isolate song of wn-reared males, we turned off the streaming white noise for approximately one hour (so as to avoid prolonged experience with hearing his own vocalizations) and recorded the song in this noise-free environment. All recorded songs were highly abnormal and were characterized by an over-abundance of noisy syllables, irregular temporal structure, and the presence of abnormally long strings of repeated syllables (for a particular exemplar, see Figure 1). While these are also characteristics of song of zebra finch males raised without a live tutor, the songs of our wn-reared males also had very few call-like notes and revealed a power spectrum that was shifted towards higher frequencies, relative to songs of both control and isolate males previously recorded in our colony. However, a larger sample size is needed for a more detailed and quantitative analysis and not in the scope of this experiment.

All surgeries were performed under Equithesin anesthesia, and neural recordings under Urethane anesthesia, and all efforts were made to minimize suffering. Detailed methods for the animal surgical procedure can be found in [26] and [27]; however, in brief, two days prior to the physiological recording experiments, birds underwent a craniotomy under Equithesin anesthesia which involved: stereotaxic positioning of the bird; removal of a small section of skin on the head; removal of top layer of the skull; adding reference points for electrode penetrations; and gluing a stainless steel post on the head with dental cement. On the day of the neural recordings, the bird was anesthetized with Urethane, after which the bird's head was immobilized in the stereotax, the lower layer of the skull and the dura were removed from the area surrounding the designated electrode location, and tungsten extracellular electrodes of resistance 1–4 MΩ (AM-Systems) were lowered into the brain. Single and multi-unit recordings of neural responses in field L were obtained in acute extracellular recordings, and body temperature was continuously monitored and adjusted.

### Stimulus design

**Figure 2:**
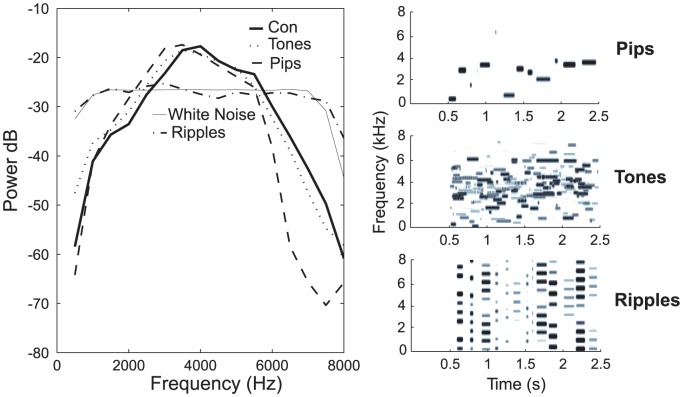
Power spectra and statistically-matched synthetic stimuli used in neural selectivity analyses. (left panel) Spectra were estimated using all the sounds used in the experiments, approximately 40 s of sound for each stimulus type. Tones and Pips ensembles were designed to match the power spectrum of conspecific song (Con) and have very similar bell-shaped spectra. Discrepancies between the Pips and the Con ensembles are due to sampling errors. White Noise and Ripples stimuli have flat power spectra between 1 and 7 kHz. (right panels) Spectrographic representation (frequencies ranging from 500 to 8,000 Hz on the *y*-axis and time in seconds on the *x*-axis) of exemplars of matched synthetic stimulus types (Pips, Tones, Ripples) used in analyzing neural responsivity and selectivity in our study. Note that the sounds in these exemplars begin at 0.5 s and last about 2 s each.

**Figure 3:**
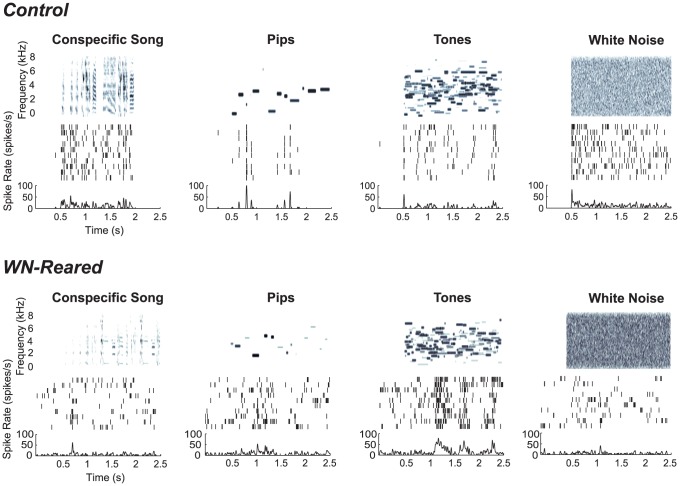
Example neural responses from a control bird and a wn-reared bird to a subset of the selectivity stimuli. Spectrographic representation of exemplars of a subset of stimulus types (Con, Pips, Tones, WN) and corresponding neural responses for 2 recording sites, one from a control adult (top) recorded in L1 and the other from a wn-reared bird recorded in L3 (bottom). Note that sound begins at 0.5 s. For the neural response, both the spike raster for 10 trials (middle) and the PSTH (denoted by spikes/s on the bottom) are shown. These examples were chosen to reflect the characteristics in the average neural responses in control adults versus wn-reared adults: the recording site from the control adult shows robust responses to Con and WN, whereas the recording site from the wn-reared adult shows decreased responses to Con and enhanced responses to Tones. In this example, as in the average data, the wn-reared recording site was more variable (Con FF = 1.16 and Pip FF = 1.40) than the control recording site (Con FF = 0.91 and Pip FF = 0.91). While these responses were chosen as illustrative of the average neural responses, we also found a wide range of response properties, including neurons in wn-reared animals that showed strong and reliable responses to song (as shown below). The spontaneous background activity was variable across units in both control and wn-reared birds but similar in rate across the two rearing conditions. To conserve space, we omitted showing the response to Ripples.

The stimulus repertoire used to probe neural selectivity consisted of natural sounds and statistically-matched synthetic sounds. The natural sound ensemble consisted of only conspecific song (Con) of 20 unfamiliar adult male zebra finches. The synthetic sound ensemble consisted of: a succession of pure tones (Pips), combination tones (Tones), spectrally modulated harmonic stacks (Ripples) and band-pass white noise (WN). Spectrograms of specific exemplars from the Con, Pips, Tones, Ripples and WN ensembles and their average power spectra are shown in Figures 2 and 3. We used 20 Pips, 20 Tones, 40 Ripples and 20 WN stimuli in all, each of 2 second duration. We played 10 presentations each of 3 different Cons, 3 different Pips, 3 different Tones, 3 different Ripples, and 2 different WN sounds for each recording site.

The synthetic stimuli were based on stimuli commonly used to characterize auditory neurons but with the additional constraint that they were designed to statistically match, on average, the power spectrum of songs (see Figure 2) as well as other parameters characterizing the syllable duration, the inter-syllable intervals and the harmonic stacks observed in the conspecific songs (see [26] for complete design details of our stimuli). In brief, Pips were a succession of pure tones (or tone pips) with similar temporal characteristics found in syllables of zebra finch song and the same overall power spectrum of song, and therefore could be thought of as the simplest (and narrowband) synthetic song that could be constructed with a series of tone pips. The frequencies of the tone pips in the Pips ensemble were derived by a random sampling of the power distribution of song, while the length of each of the tone pips and the inter-pip silences were drawn from a Gaussian distribution that approximated the distribution of the length of song syllables (95±66 (SD) ms) and inter-syllable silences (37±25 (SD) ms). The onset and offset ramp of each tone pip was a 25 ms cosine function, loosely matching the amplitude envelope of song syllables.

Since the Pips lacked the multi-band or broadband quality of sound characteristic of zebra finch song, we designed the Tones ensemble to be a broadband extension of the Pips ensemble. The Tones were synthesized by adding 20 different Pips sounds together and normalizing the result to maintain the overall power spectrum of song, and could be considered in this sense as sparse-colored noise in reference to their song-like power spectra. The range of intensity in any narrow frequency band in the Tones ensemble was similar to that found in song.

Zebra finch song contains many song syllables composed of harmonically related frequency components, which was lacking in our Tones ensemble. For this reason, we designed the Ripples ensemble composed of slow-varying harmonic stacks along the frequency axis. The fundamental of the harmonic stacks in our Ripples ensemble was chosen from a Gaussian distribution with a mean of 700±100 Hz to match the range of fundamental frequencies in the harmonic stacks found in zebra finch song. Similar to the Pips ensemble, the duration of each harmonic stack and the inter-stack interval had the same mean and standard deviation as zebra finch song syllable and inter-syllable duration. The overall power spectrum of the Ripples ensemble was flat from 700 Hz to 8 kHz.

Finally, White Noise was used as the classic unstructured and broadband synthetic stimulus that was also the auditory rearing environment of the experimental animal. In particular, our White Noise stimuli were band-passed from 16 Hz to 8 kHz, with an upper limit of 8 kHz used in order to keep in line with the 8 kHz cutoff of the other stimuli in our ensembles, and the overall power of White Noise, while flat, was matched to those of song and the other synthetic stimulus ensembles.

The stimulus presentation order was randomized per trial, and a random inter-stimulus interval with a uniform distribution of 7 to 8 seconds was used. The volume of the speaker was set to deliver song at peak levels of 80 dB SPL. Two seconds of spontaneous spiking were recorded both before and after the stimulus presentation. These recording parameters (used for our selectivity analysis) were identical to those used in the recordings reported in [26] since our goal was to directly compare the average neural responses (and average neural selectivity) obtained from our wn-reared birds to those obtained from control birds [26].

In addition to the ensemble of songs and synthetic sounds to probe neural selectivity for natural sounds, we also played 10 exemplars of modulation-limited noise (ML-Noise) and an additional 20 exemplars of zebra finch song (Con), 10–14 presentations of each exemplar, for a subset of the recording sites (n = 27) for which STRFs, Gamma Information, and ensemble Mutual Information values would be calculated. ML-Noise had uniformly sampled spectral-temporal modulations that contained the modulations found in song as well as modulations absent in song. More specifically, ML-Noise is white noise for which we low-passed the log amplitude envelope modulations to temporal modulations <50 Hz and spectral modulations <2 cycles/kHz. To maximize the number of recorded sites, these additional stimuli were only presented to the subset of neurons that exhibited a reliable time-varying auditory response across trials. This ensemble of Con and ML-Noise stimuli were presented at a peak intensity of 70 dB sound pressure level. A random interstimulus interval with a uniform distribution between 4 and 6 s was used. These recording parameters were identical to those used in the recordings reported in [33] since our goal was to compare information values and STRFs obtained from our wn-reared birds to those obtained from control birds [33].

### Electrophysiology and experimental protocol

Neural recordings were conducted in a sound-attenuated chamber. Single and multi-unit spike arrival times were obtained by thresholding the extracellular voltage trace with a window discriminator. The multi-unit data were obtained with a high window threshold relative to the noise level and consisted mostly of a small cluster of units (2–4 neurons, as ascertained by visual examination of the saved spike waveform shape), while single units were classified only upon meeting the following criteria: possessing a high signal-to-noise ratio in the recordings (amplitude signal-to-noise ratio >5), monitoring the shape of the triggered action potentials on a digital oscilloscope with trace storage, and calculating the distribution of inter-spike intervals post hoc. All inter-spike distributions from the visually determined single units showed a signature depression between 0 and 1 ms from postspiking inhibition.

Since our goal was to directly compare the neural selectivity results from the wn-reared birds to those of normal adults (controls) from [26], we followed the protocol from that study: we systematically recorded from a large area of the field L complex both rostro-caudally (900 to 1500 microns rostral of the y-sinus) and medio-laterally (1050 to 1800 microns from the midline) and systematically sampled neuronal sites every 100 microns during each electrode pass. The position of the electrode was varied from its 100 micron step position if this repositioning allowed for better isolation of a single unit. For our Gamma Information, ensemble Mutual Information, and STRF analyses, we used only single unit recordings for which we were able to obtain enough spikes in response to either Con or ML-Noise to estimate reliable STRFs, and used the same criteria as in our prior STRFs mapping [33]. Since most recording sites in these experimental birds did not exhibit strong time-varying responses to complex sounds, we were only able to obtain STRF data for a subset of our single unit recording sites (n = 27), which was also used for our Gamma Information, and ensemble and redundancy calculations. Finally, electrode penetrations in a given bird were at least 300 microns apart. Between one to two electrode penetrations were achieved per bird. At the end of each electrode penetration, two electrolytic lesions (100 µA for 5 s each) 300 microns apart were made (one of which was made 400 microns after the last recording site and in regions well below the auditory forebrain in the case of the first recording pass, or at the last recording site itself for the last recording pass). The lesions aided in the later reconstruction of the recording sites, while the creation of two lesions aided in calibrating our depth measures. We did not observe any differences in response properties between recordings prior and after lesions.

### Histology and anatomical reconstructions

At the end of the electrophysiological recordings, the bird was deeply anesthetized with 0.15 cc of Equithesin and transcardially perfused with 0.9% saline, followed by 3.7% formalin in 0.025 M phosphate buffer. The skullcap was removed and the brain was postfixed in 30% sucrose and 3.7% formalin to prepare it for histological procedures. The brain was sliced parasagittally in 40-µm-thick sections using a freezing microtome, and alternating brain sections were stained with both cresyl violet and silver stain, which were then used to visualize electrode tracks and electrolytic lesions.

Recording sites were reconstructed by measuring both the distance from the entry of the electrode pass to the lesion and the distance between successive lesions and comparing these distances in microns with the reading of our independently calibrated microdrive used during the experiment. The sites were then reconstructed with the aid of the experimental log, containing microdrive-measured distances between subsequent sites, as a reference. Using well-known anatomical landmarks such as the pallial-subpallial lamina (LPS) and differences in cell size, shape, and density as described in the literature [34], neural sites were then assigned to either the thalamo-recipient subdivision L2 (L2a or L2b), or sub-regions L1 and L3. Any recording sites that were determined to be on the border of a subfield were assigned to that subfield (within less than 50 microns). L2a and L2b were the most readily distinguishable subfields based on cell shape and size. Subfield L1 was defined as the area that was dorsal to the boundary of L2 and below the lamina that divides the nidopallium and the mesopallium. Subfield L3 was defined as the area below the ventral boundary of L2 within the nidopallium. We were not able to distinguish a boundary between subfield L3 and subfield L. All the ventral recording sites were assigned to L3 with that caveat in mind.

### Data Analysis

#### Response Strength, Neural Selectivity, and Fano Factor Analysis

Neurons from both control and wn-reared birds were first classified according to whether they were responsive or not. To be classified as responsive, a unit had to have an average firing rate for either Pips, Tones, Ripples, WN, or Con that was significantly different from its pre-stimulus spontaneous rate as assessed by two-tailed paired t-tests. The null hypothesis was rejected when either one of the following two situations arose: when normalized responses to at least one stimulus class yielded p<0.01 (corresponding to a significance value of α = 0.05 after the Bonferroni correction for 5 comparisons); or when normalized responses to two stimulus classes each yielded p<0.05 (corresponding to a significance value of α = 0.025 obtained from the binomial distribution and finding 2 responses out 5 below α).

All responsive sites were given a z-score for each stimulus, which characterizes the normalized difference between the stimulus-evoked mean firing rate and that of the two second background activity preceding the stimulus. The z-score is calculated as follows:
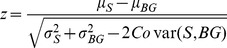
where μ_S_ is the mean response during the stimulus, μ_BG_ is the mean response during the background, σ_S_^2^ is the variance of the response during the stimulus, and σ_BG_^2^ the variance of the response during baseline. In calculating a recording site's z-score to a particular stimulus type, responses were averaged to all presentations for that particular stimulus type for the site. For instance, a site's response to three exemplars of Con was averaged together when calculating that site's single z-score measure to conspecific song. We chose to use z-scores as a metric for stimulus-evoked response strength over simply reporting firing rates since z-scores take into account differences in background firing rates across different neurons. We further classified units as ‘stimulus-excited’ if the responsive units had a significant positive z-score to any of the stimuli.

The selectivity of each unit for one stimulus class over another stimulus class was quantified using the psychophysical d' measure. In neurophysiological research, the d' measure is used to quantify pairwise response differences that might otherwise go undetected in the average response across many units. The d' measure for the neural discriminability between two stimuli A and B at the single neuron/site level is calculated as:
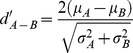
where μ_A_ and μ_B_ are the mean responses to stimulus A and B, respectively, and σ^2^ is the variance of the response. If the d' value is positive then stimulus A elicited a greater response, if it is negative then stimulus B elicited a greater response. d' values ∼ 0 indicate no difference in the response evoked by the two stimuli. The d' measure is sensitive to the sign of the difference in magnitude of the absolute responses and will therefore give negative values when stimulus A elicited a greater inhibition than the inhibition obtained to stimulus B. Since the average neural response was greatest for Conspecific song in field L neurons of control birds [26], we decided to use song as our standard comparator in d' comparisons for our wn-reared birds as well.

We also analyzed the neural variability of wn-reared and control stimulus-excited recording sites by computing the Fano Factor (FF) of time-varying mean firing rates across trials in response to Con (the most natural stimulus in our stimulus ensemble) and to Pips (the most simple stimulus in our stimulus ensemble). The FF is the ratio of the variance of the spike rate over its mean. The time-varying rates were obtained by convolving spike trains with 30 ms rectangular windows. The variance and mean were estimated for each time point over the 10 spike trials in response to the stimulus class to estimate a time-varying FF. The FF was then averaged across time and across songs (or Pips) for each neuron. To obtain reliable measures, the FF was only calculated for stimulus-excited recording sites that had a background-subtracted spike rate >1 spike/s in response to the stimulus class.

#### STRF Calculation

A regularized and normalized reverse correlation analysis was used to determine the relation between the stimulus and responses. This analysis yields the Spectro-Temporal Receptive Field (STRF), a model of a neuron's auditory tuning properties. The STRF calculation entailed three steps. First, the log-intensity spectrogram of the sound stimulus (i.e. a sample of song) was cross-correlated with the time-varying mean response to that stimulus (averaged across the 10 to 14 trials) to obtain the spike-triggered average. Second, the spike-triggered average was normalized by the autocorrelations of the stimulus. Third, a regularization-cross validation procedure was used to effectively minimize the number of parameters fitted in the STRF estimation. Once the STRF was obtained, it was validated on data that were not used in the STRF calculation. The similarity between the predicted response and the actual response, measured using noise-corrected correlation coefficients (CCratio), provided a measure of how well the STRF captures the tuning of a neuron [28]. Neurons for which the STRF gave poor predictions (CCratio<0.2) were excluded from the single neuron discrimination analyses and ensemble neuron discrimination analyses, for both controls and wn-reared neurons (see below). Detailed descriptions of this STRF methodology are found in [35], [6], [36], [33]. STRF estimation and validations were done using STRFPAK, a Matlab toolbox developed by the Theunissen and Gallant labs at UC Berkeley (strfpak.berkeley.edu).

We also computed the pairwise similarity of STRFs by estimating the correlation coefficient between two STRFs after allowing shifts in frequency and in latency. This pair-wise measure of similarity is called the similarity index (SI). We calculated the SI between each of the neurons in the wn-reared data set and all the neurons in the control data set. The control neuron that yielded the highest SI was taken as the best-match. We used this ‘matched SI’ for part of our Gamma Information analysis below, and only neurons with a max SI of 0.5 or greater were used for the ‘matched SI’ Gamma Information analysis. The threshold of 0.5 was chosen because it corresponded to the minimum SI found between two neurons belonging to the same functional class in our control data set.

#### Single neuron discrimination

*Gamma Information:* To directly evaluate the neuron's ability to efficiently represent different song (Con) stimuli or different ML-Noise stimuli, we estimated measures of single neuron information, and measures of multi-neuron information and redundancy. First, we calculated the discriminability of single neurons by estimating the mutual information (MI) between the sound stimulus and the neural response of single units using a framework that involved modeling the neural spike patterns as an inhomogeneous Gamma process. This information calculation involved estimating the time-varying mean firing rate and the order of the gamma process that best matched the data (see [28] for more details). A model Gamma neuron with these fitted parameters was then used to generate sufficient model spike trains (500 or more) in order to calculate the MI using the direct approach [37].

#### Ensemble neural discrimination

Finally, we also estimated the mutual information and redundancy for small ensembles of neurons in encoding Con and ML-Noise. It is computationally problematic to calculate ensemble MI from spike patterns due to the large number of dimensions of the probability densities of interest. To address this challenge, we adopted a decoding/classifier approach that allowed us to transform ensemble neural responses into a guess of the stimulus that was presented. Upon decoding the spike trains, we obtained a confusion matrix representing the joint probability of the stimulus and the best guess from the response.

The decoding procedure involved generating a spike pattern template for each neuron in response to each stimulus from a fraction of the responses, 9/10 trials in our case. These templates were obtained by convolving each spike train with a decaying exponential [38]. The remaining spike train was left to be decoded and this procedure was repeated for all stimuli and jackknifed over all trials. The decoding involved calculating the Euclidian distance (also called the Van Rossum or VR distance) between the templates and the test spike train. The decoded song was simply the one that corresponded to the minimum VR distance. The ensemble VR distance was taken to be the sum of the individual VR distances after normalization by the average distance between templates for the corresponding neuron. This normalization yielded a weighted average in which neurons that carried more information had larger weights than neurons that carried less information.

We then estimated the mutual information between the stimulus (s) and response (r):
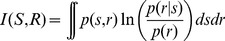
In this equation, s represents the stimulus identity (e.g. the particular song) and r the neural response. Optimally, this neural response would be a long vector corresponding to the spike raster: the presence and absence of spikes during the stimulus presentation. In practice, it is very difficult to estimate the probability density functions of such response vectors (but see [37]) and even more so when ensemble responses are considered. Our decoding method allowed us to address this issue by transforming these multi-dimensional neural responses vectors into uni-dimensional distances: a neural response vector was reduced to a VR distance to a particular template (a single number with units of (spikes/s)^2^). The conditional distribution of p(r|s) was then replaced by the distribution of distances between the response trial for a song and the template for that same song (the self-distance), and the unconditional distribution of p(r) was replaced by the distribution of distances between the response trial and all templates.

We have previously shown that this estimate of MI is a lower bound for the actual MI and that, as the number of neurons increase, this lower bound can grossly underestimate the actual MI. A tighter upper bound can be found by applying what we have called the anthropic correction (see [39] for detailed methods for estimating the ensemble information, for the statistical properties of the anthropic estimate, and for a validation of this approach by comparison with other estimates of the MI). The anthropic correction was obtained by excluding the self-distances in the distribution of all distances. In other words, the distribution of all distances was replaced by the distribution of distances to other songs. These distributions of distances were also well fitted by normal distributions. With these approximations, the anthropic MI is given by:
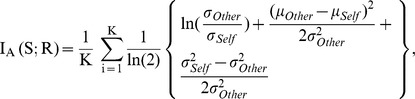
where μand σ are the mean and standard deviation of the distribution of distances and K is the number of stimuli (here K = 20 songs or K = 10 ML-Noise stimuli). This estimate of the mutual information can be obtained for a single neuron or an ensemble of neurons. Here, we calculated ensemble information for groups of M = 2 to 10 neurons. The redundancy in the neural representation can then be calculated by comparing the MI obtained from a single neuron m, I(S;R_m_), to the MI obtained from the ensemble I(S;R_1_,R_2_,…,R_M_):
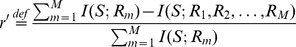



*This measure of redundancy is 0 if neurons encode independent information, 1 if neurons are completely redundant and can be negative if the neural code is synergistic.*

## Results

### An overall decrease in response strength to song but not simple sounds

We recorded neural responses from 192 responsive recording sites from 14 wn-reared birds, of which 177 (92.2%) sites were defined to be stimulus-excited (see Methods). 113 (63.8%) of these stimulus-excited sites were single units, whereas 64 (36.2%) were multi-units. Since we found no differences in the data obtained from our wn-reared males and females, we pooled the data from males and females for all our analyses. We compared these responses to those obtained from 200 responsive recording sites from 24 control birds, of which 175 (87.5%) sites were classified as stimulus-excited [26]. 64 (36.6%) of these stimulus-excited sites were single units and 111 (63.4%) were multi-units. There was no statistical difference in the percentage of stimulus-excited responsive units between the two rearing conditions (Chi-Square test for independence). Similarly, the overall background rate (bg), pooled across all sub-regions and across single and multi-unit recording sites, was not statistically different between the two rearing conditions (control bg = 3.5 spikes/s, wn-reared bg = 3.97 spikes/s, t(347) = −1.24, p = 0.22). We also examined the background rates of single and multi-units separately, in addition to examining each sub-region separately, and found both single unit recordings and multi-unit recordings had statistically similar background rates in L1 and L2, as did the multi-unit recordings in L3. Only in L3 did we find higher background firing rates for single unit recordings in wn-reared animals (control = 2.41 spikes/s, wn-reared = 3.62 spikes/s, t(92) = −2.18, p = 0.03).

Because a multiple linear regression using unit type (single vs multi), stimulus type and rearing condition to predict z-scores did not show any interaction effects between unit type and stimulus type or unit type and rearing condition, we merged the responses from single and multi-units for all analyses based on normalized rates: these include analyses of neural responsivity (z-score), selectivity (d′) and variability (FF). However, for the STRFs, the single-neuron and ensemble information analyses, only single units were used in both the wn-reared and control case (see Methods for details).

**Figure 4:**
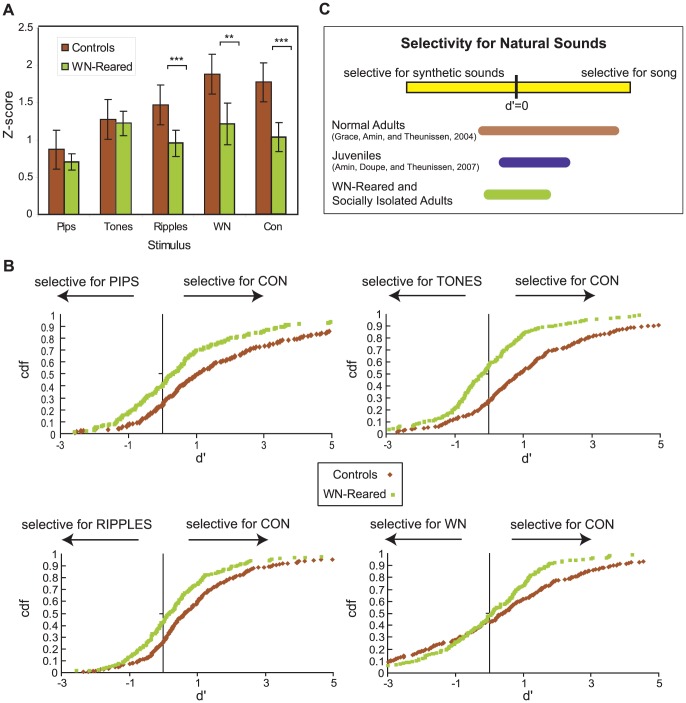
Neural responsivity and selectivity, as measured by z-scores and d′ values respectively. A. Comparison of control and wn-reared mean z-scores for all the stimuli used in the selectivity analysis (Con, Pips, Tones, Ripples, and WN). Average z-scores for all stimulus-excited responsive units show that responses to the more complex stimuli of Con, Ripples, and WN are reduced in wn-reared birds compared to control birds. Error bars represent 2 SEs. B. Cumulative distribution functions (cdf) of d′ values for the Con-Pips, Con-Tones, Con-Ripples, and Con-WN comparisons for both controls and wn-reared adults. Selectivity analyses (and the resulting cumulative curves) show the greatest divergence in rearing conditions for the Con-Tones, followed by the Con-Pips and Con-Ripples comparisons. C. Working model of the development of the neural selectivity for natural sounds such as song (d′>0 for song as compared to synthetic sounds), as a function of development and natural rearing environments, in the auditory system of songbirds. The bars in this schematic summarize the distributions of d′ values obtained from our data from control adults, juveniles, and wn-reared and socially-isolated adults.

We found the neural responses of a majority of the neurons in wn-reared birds were significantly altered compared to the controls. Figure 3 shows two example neurons: one from a control adult and the other from a wn-reared adult in response to Con and a subset of the matched synthetic sounds (Pips, Tones, and WN). Typical of the average wn-reared neuron, the firing rate across trials is more variable and the response to Con in this example is decreased relative to Pips and Tones, contrary to what is observed in the control example. The average response strength, as defined by z-scores, for the stimulus-excited sites shown in Figure 4a, illustrates that in wn-reared animals Tones elicited the largest response followed by WN, Con, Ripples, and Pips (wn-reared: F(4, 880) = 4.92, p<0.001), whereas Con and WN elicited the most spikes in the control group (controls: F(4, 869) = 9.45, p<0.001). Moreover, the average response to the simpler synthetic stimuli Tones and Pips in wn-reared animals were similar to the controls (Tones: Δz = −0.05, t(320) = −0.3, p = 0.69; Pips:Δz = −0.16, t(290) = −1.6, p = 0.10), consistent with the fact that wn-reared birds had normal intensity-response curves. We further examined the coarse frequency versus intensity tuning of the wn-reared birds by plotting response maps (frequency vs. intensity) from the pure tone responses that we obtained from the Pips stimuli and found these 2-d curves similar to those of control birds (plots not shown). However, there was an overall decrease in average z-scores to the more complex stimuli of Con, Ripples, and WN in the wn-reared condition, with the greatest decrease in response to Con (Con: Δz = −0.73, t(313) = −4.39, p<0.001; Ripples: Δz = −0.51, t(304) = −3.32, p<0.001; WN: Δz = −0.67, t(330) = −2.98, p = 0.003). Finally, z-score distributions (pooled for the five stimulus types) were significantly different between the two rearing conditions: F(1,1757) = 33.76, p<0.001. The z-score measure of response strength, which takes into account both the background and stimulus-evoked rates, was largely affected by differences in evoked firing rates in the two rearing conditions (since background rates were similar for both conditions, as mentioned above). For instance, we found statistically similar firing rates in response to Pips in the two populations (control = 8.78 spikes/s, wn-reared = 7.58 spikes/s, t(681) = 1.74, p = 0.08) but a statistically significant decrease in response to Con in wn-reared birds (control = 12.04 spikes/s, wn-reared = 9.03 spikes/s, Δr = 3 spikes/s, t(332) = 2.4, p = 0.015). Therefore these analyses reveal an overall *depressed* response in wn-reared birds to song and other complex stimuli.

Although the average wn-reared neuron had an overall decrease in response to song, there was a subset of neurons that were similar to the average control neurons in that they had a similar and substantial neural response to conspecific song, and were able to phase-lock reliably to sound stimuli. We will introduce and analyze this subset in the single and ensemble neuron discrimination below.

### Overall neural selectivity diminished for song versus synthetic sounds

To directly compare the responses to conspecific song with those obtained in response to our matched synthetic sounds, we quantified the selectivity of any given unit by calculating a d′ value: the normalized pairwise difference between the neural responses to two stimuli being compared. The average d′ value in control birds was significantly greater than 0 for Con over Pips, Tones, and Ripples: Con-Pips (mean d′ = 2.04, t(241) = 9.65, p<0.0001); Con-Tones (mean d′ = 1.34, t(240) = 8.77, p<0.0001); and Con-Ripples (mean d′ = 0.97, t(241) = 8.99, p<0.0001). The mean d′ value for Con over WN also showed a non-significant positive trend in favor of Con (Con-WN mean d′ = 0.28, t(238) = 1.51, p = 0.13). These analyses further showed that field L neurons of control zebra finches were selective for conspecific song relative to a subset of our matched synthetic sounds. The strongest preference for Con was over Pips, followed by Tones and then Ripples.

This selectivity for song however changed dramatically in the wn-reared birds. The average d′ value was significantly greater than 0 only for Con over Pips and Ripples, and the effect size in those comparisons was reduced by more than 50%: Con-Pips (mean d′ = 0.82, t(191) = 5.08, p<0.0001); Con-Tones (mean d′ = −0.02, t(191) = −0.24, p = 0.80); Con-Ripples (mean d′ = 0.41, t(191) = 3.76, p = 0.0002); and Con-WN (mean d′ = 0.08, t(191) = −0.55, p = 0.57).

The average d′ values were significantly different between wn-reared and control birds for the Con-Pips (Δd′ = −1.21, t(432) = −4.35, p<0.0001), Con-Tones (Δd′ = −1.37, t(431) = −6.88, p<0.0001), and Con-Ripples (Δd′ = −0.55, t(431) = −3.56, p = 0.0004) comparisons, but not for the Con-WN comparison (Δd′ = −0.36, t(429) = −1.47, p = 0.14).

The cumulative distribution plots in Figure 4b are useful for illustrating the effect size. In the Con vs. Pips comparison and the Con vs. Ripples comparison, approximately 20% of the neural recording sites from the control group had a greater mean response to synthetic sounds than to songs, whereas about 40% of the sites from the wn-reared group had a greater response to the synthetic sounds. The effect size for Con vs. Tones was particularly dramatic: ∼25% of the recording sites from the control condition group were selective for Tones, whereas ∼60% of the sites from the wn-reared group preferred Tones to Con. These results thus indicate that field L neurons are sensitive to acoustic environmental manipulations, to the point where firing-rate-based tuning to natural sounds, such as song, is substantially affected when natural spectro-temporal acoustic patterns are missing from the environment. The schematic in Figure 4c summarizes the results of the selectivity analyses performed here in wn-reared birds and compares these results to the neural selectivity observed in control birds [26] and also in normal young birds [27]: the reduced selectivity found in wn-reared adult birds is reminiscent of the reduced selectivity found in juveniles.

### Subfield L2 of wn-reared birds similar to controls in overall responsivity and selectivity for song

**Figure 5:**
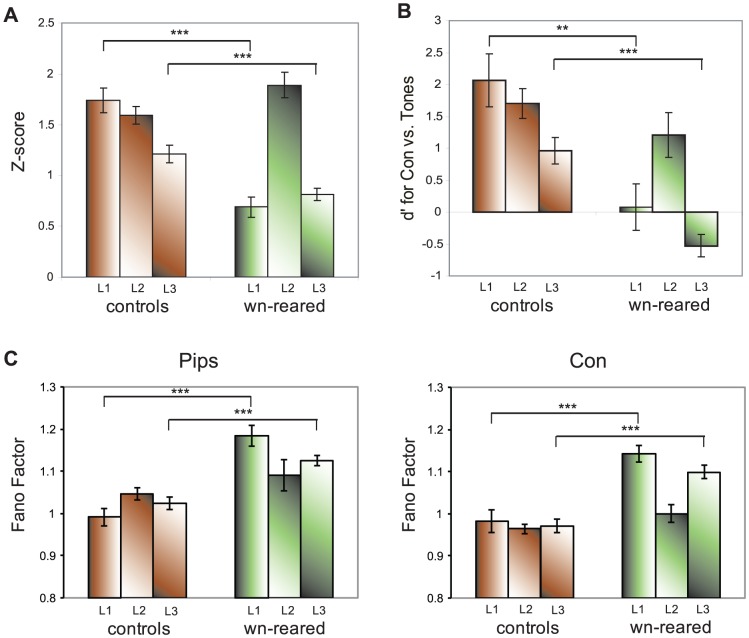
Neural responsivity, selectivity, and reliability in subfields L1 (∼Layer III), L2 (∼Layer IV), and L3 (∼Layer V). A. Comparison of control and wn-reared mean z-scores (pooled over all five stimuli: Con, Pips, Tones, Ripples, and WN) broken down by subdivisions in field L (as assessed by histological analysis). Average z-scores for all stimulus-excited responsive units show that responses to the more complex stimuli of Con, Ripples, and WN are reduced in L1 and L3 in wn-reared birds compared to control birds. Error bars represent 2 SEs. B. d′ values for the Con-Tones comparison for controls and wn-reared adults broken down by subdivisions in field L. Selectivity analysis shows noise-rearing had the greatest effect on subfields L1 and L3. Error bars represent 2 SEs. C. Fano factor for Pips (left) and Tones (right) for controls and wn-reared adults broken down by subdivisions in field L. Noise-rearing decreased the response reliability for neurons in subfields L1 and L3. Error bars represent 2 SEs.

We also examined whether there are any responsivity or tuning differences between the different subfields of field L, namely subfields L1, L2, and L3 across the rearing conditions. Subfield L2a (homologous to Layer IV in A1 in mammals) is the principal recipient of thalamic input from Ovoidalis (the homolog of the mammalian ventral medio-geniculate body (MGv)), which then projects superficially to L1 (∼Layer III) and Caudal Mesopallium (CM) (∼Layers I–II). The CM in turn sends recurrent projections back to L1 and L2a, and many axons appear to continue into deep subfield L3 (which along with other auditory telencephalic nuclei Nd and Aivm functionally make up deep Layers V–VI) (see [40]). We hypothesized that if there was a population of neurons in wn-reared birds that was similar in tuning for complex natural sounds relative to synthetic sounds as those found in control birds, then most would likely reside in the thalamorecipient L2 sub-region, the lowest information processing level in cortex. In control adults, L1 and L2 were most responsive (z-score distributions pooled across all five stimuli: L1 mean z = 1.73, std = 0.16; L2 mean z = 1.58, std = 0.08)), followed by L3 (mean z = 1.19, std = 0.09), and an ANOVA confirmed the differences amongst sub-regions (F(2, 871) = 6.38; p = 0.0018). However, in the wn-reared group, L2 was by far the most responsive (mean z = 1.88, std = 0.08), followed by L3 and L1 (L3 mean z = 0.81, std = 0.05; L1 mean z = 0.68; std = 0.09), and the ANOVA also showed a significant difference amongst subfields (F(2,852) = 66.17, p<0.0001). We also performed a 3-way ANOVA on z-scores for effects of stimulus class, sub-region, and rearing condition, and found a significant main effect for all three variables (stimulus class: F(4,1639) = 9.53, p<0.0001; sub-region: F(2,1639) = 34.54, p<0.0001; rearing condition: F(1,1639) = 23.14, p<0.0001), and also found significant 2-way interactions between stimulus class and sub-region (F(8,1639) = 2.10, p = 0.0322), sub-region and rearing condition (F(2,1639) = 21.09, p<0.0001), but not for stimulus class and rearing condition (F(4,1639) = 2.02, p = 0.089). Next we tested for sub-region differences in responsivity between wn-reared and control birds (and applied a Bonferonni Correction for the 3 comparisons), and found that in accordance to our initial hypothesis only wn-reared L2 was similar to controls (Δz = 0.29, t(602) = 1.95, p = 0.15), whereas wn-reared subfields L1 and L3 did not demonstrate the degree of responsivity found in controls (L1: Δz = −1.04, t(293) = −6.65, p<0.0001; L3: Δz = −0.39, t(703) = −3.77, p = 0.0006 (Bonferroni-adjusted)) (see Figure 5A).

As expected from the sub-regional differences in z-scores above and also the overall selectivity analysis above, the largest d′ differences between wn-reared and control birds were found for the Con-Tones comparison in the L1 and L3 subfields (and highlighted in Figure 5B; L1: Δd′ = −1.98, t(65) = −3.59, p = 0.0018; L3: Δd′ = −1.48, t(182) = −5.54, p<0.0001), followed by the Con-Pips comparison in L1 and L3 (L1: Δd′ = −1.54, t(65) = −2.54, p = 0.0402; L3: Δd′ = −1.55, t(182) = −3.91, p<0.0001), and finally the Con-Ripples comparison in L3 (Δd′ = −0.70, t(65) = −2.96, p = 0.0102 (all p-values were Bonferroni-corrected for the 3 subfield comparisons)). All other selectivity comparisons, broken down by subfields, did not achieve statistical significance. We discuss below the significance of thalamorecipient L2 having comparable song-selective responses in noise-reared and control birds, versus the experience-dependent song-selective development in extrathalamic subfields.

#### An increase in overall neural variability

In addition to firing-rate-based tuning, as described above for responsivity and selectivity measures, we also checked for differences in firing patterns, such as neural variability, across rearing conditions. Cross-trial Fano Factor (FF) values computed for both control and wn-reared stimulus-excited sites in response to both Con and Pips showed a significant difference in FF (*Con*: controls: average FF = 0.969, stderr = 0.009; wn-reared: average FF = 1.081, stderr = 0.011; ΔFF = −0.11, Effect Size (or ES) = −0.92, t(286) = −7.70, p<0.0001; *Pips*: controls: average FF = 1.029, stderr = 0.009; wn-reared: average FF = 1.128, stderr = 0.011; ΔFF = −0.098, ES = −0.75, t(281) = −6.24, p<0.0001), illustrating that the wn-reared neurons were more variable and less able to phase-lock to the sound stimulus. Note that although the change in FF may appear small, it is in fact a large effect size (ES) given the restricted range that FF can take and the consistency of this measure across neurons in each population. Since FFs can vary with mean firing rates (and by extension z-scores), we also estimated the significance of the rearing condition in a general linear model framework that included both rearing condition and z-scores as regressors. Both coefficients were significant as sites with higher z-score values had on average lower FF (data not shown). Nonetheless, the effect of rearing condition on FF after taking z-score differences into account remained highly significant (*Con*: F(1,285) = 31.39, p<0.0001; *Pips*: F(1,280) = 34.18, p<0.0001).

We also tested for differences in FF values amongst field L sub-regions, and in line with the sub-regional z-score analysis, L1 and L3 but *not* L2 revealed FF differences between control and wn-reared stimulus-excited sites in response to Pips and Con (see Figure 5C) (*Pips*: *L1*: t(48) = −5.60, p<0.0001; *L2*: t(101) = −1.30, p = 0.19; *L3*: t(123) = −5.14, p<0.0001; *Con: L1:* t(50) = −4.84, p<0.0001; *L2*:controls: t(101) = −1.63, p = 0.10; *L3*: t(124) = −5.6, p<0.0001). Upon taking into account the effect of z-scores on FF in a general linear model, we found that, as in the overall analysis, FF values decreased with z-scores in all sub-regions but the effect of rearing condition on FF remained highly significant only for sub-regions L1 and L3 (*Con: L1:* F(1,49) = 12.89, p = 0.0008*; L2:* F(1,100) = 1.96, p = 0.165; *L3:* F(1,123) = 13.5, p = 0.0004; *Pips: L1:* F(1,47) = 21.31, p<0.0001; *L2:* F(1,100) = 4.87, p = 0.088; *L3:* F(1,122) = 21.6, p<0.0001).

#### STRFs derived from noise-exposed field L neurons are functionally similar to those derived from control birds but predict song less reliably

We found that field L of wn-reared birds had a smaller number of neurons for which we could reliably estimate STRFs from their responses to song. This result was likely a consequence of the decrease in response strength (z-score) and an increase in variability (FF) described above, especially in subfields L1 and L3. In our systematic sampling of the field L complex, we were able to obtain STRFs from only 27 single neurons (out of 113 single units and 192 responsive sites in all) in field L of our 14 wn-reared birds (1.93 units/bird). Other single units did not meet the minimum criteria for STRF reliability (see Methods). Of these 27 single neural sites, a large percentage (∼74%) were found in the L2 sub-region (n = 15) or on the L2 border (n = 5), while only a small number belonged to L1 (n = 2) and L3 (n = 5). On the other hand, we estimated 137 STRFs from single units in 35 control animals (3.91 units/bird) that were distributed across all sub-regions [33].

It should be noted that this subset of wn-reared neurons shared similar properties to the neurons in control birds in that they were able to phase-lock to the stimulus, and their response to song was robust, compared to matched synthetic sounds (d′ values for this wn-reared subset: Con-Pips (mean d′ = 3.95, t(25) = 8.37, p<0.0001); Con-Tones (mean d′ = 1.71, t(25) = 5.15, p<0.0001); Con-Ripples (mean d′ = 1.60, t(25) = 4.88, p<0.0001); and Con-WN (mean d′ = −0.63, t(25) = −0.83, p = 0.41)). Thus this subset of neurons was *different from the rest* of the population of wn-reared neurons that were on average less responsive to song. This result is not surprising given that this subset of neurons were largely from the L2 sub-region and as described above we found similar response rates and FF values for control and wn-reared animals in that sub-region. In the same vein, we also did not find any systematic differences in functional STRF types between the two rearing conditions (e.g. similar distributions of SIs were found for control and wn-reared neurons). Similarly, once the STRFs were classified into functional groups as in [33], the percentages of neurons classified as Narrowband (13/27 or 48.2% vs 25.5% control), Broadband (7/27 or 25.9% vs 29.9%), Wideband (0/27 or 0% vs 8.8% control), Offset (2/27 or 7.4% vs 5.1% control), Hybrid (1/27 or 3.7% vs 7.3% control), and Complex/Unclassified (wn-reared: 4/27 or 14.8% vs 23.4% control) were not statistically different in each condition (χ^2^ = 8.25, df = 5, p = 0.14), with the caveat that the small numbers of STRFs in the wn-reared group (n = 27) reduces the power of this statistical test and does not allow us to rule out small differences in percentages. Nevertheless, we subsequently show that this subset of neurons was *different from control neurons, including matched control neurons*, in that they encoded information for noise better than they encoded information for song, as we show in greater detail here with predicted responses and below with information analyses.

For each rearing condition, we estimated how much of the neural response can be explained with the linear STRF model by calculating an adjusted correlation coefficient (CCratio) between the predicted response and actual response spike rate in response to both Conspecific song and ML-Noise. In this analysis, we used the STRF estimated in response to song to predict responses to new song and the STRF estimated from ML-Noise responses to predict responses to new samples of ML-Noise. If neurons were driven into a higher-rate and more-linear mode when processing behaviorally-relevant sounds, one would expect the quality of predictions to be greater for Con than ML-Noise. This was indeed the case for control birds (CCratio = 0.53±0.011 (stderr) for Con; CCratio = 0.47±0.013 for ML-Noise; t(224) = 3.33; p<0.001), but the opposite trend was observed in wn-reared birds (CCratio = 0.43±0.027 for Con; CCratio = 0.51±0.034 for ML-Noise; (t(52) = −1.74, p = 0.08). We also found significant differences in the quality of song prediction between the two conditions (t(138) = 3.52, p<0.001), with the subset of wn-reared neurons predicting song worse than neurons from control adults. A 2-way ANOVA also showed a significant interaction between rearing condition and stimulus type (F(1,276) = 10.37, p = 0.0014). These findings help explain some of our single neuron Gamma Information analyses below.

#### Gamma Information greater for ML-Noise in single neurons

We estimated the mutual information (MI) between the sound stimulus (Con and ML-Noise) and the neural response of single neurons using a framework that involved modeling the neural spike patterns as an inhomogeneous Gamma process. To this end, we used the responses from the two rearing conditions that had both Con and ML-Noise stimuli presented to them and for which we also derived STRFs (n = 113 for the control condition and n = 27 for the wn-reared condition). Although these two populations were similar in that they both consisted of neurons that responded reliably to song and had similar selectivity properties and types of STRF tuning, the subset of wn-reared neurons (n = 27) represented a smaller fraction of the responsive neurons in field L than the 113 neurons in the control case and also consisted of a relatively larger proportion of L2 neurons than our representative control neurons. We therefore also computed Gamma Information values for a ‘matched subset’ of 27 control neurons that were most similar in tuning to the 27 wn-reared neurons. This second set of control neurons, the ‘matched control group’, was chosen by correlating the STRFs of each wn-reared neuron with each of the STRFs in the control data set and choosing the match that yielded the highest SI (see Methods).

**Figure 6:**
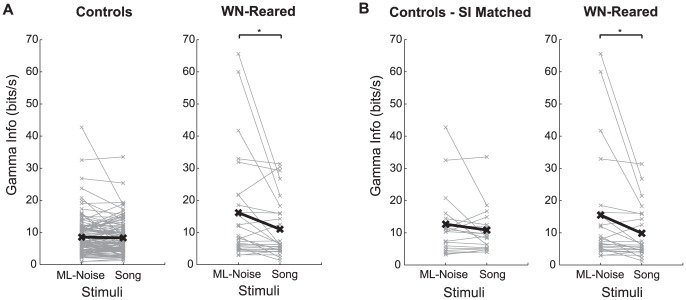
Single neuron information values greater for ML-Noise than song in wn-reared condition. A. Pairwise difference in Gamma Information rates (bits/s) for Song vs. ML-Noise estimated for single neurons for control birds (left) and wn-reared birds (right). Neurons in the auditory forebrain of wn-reared birds encode for ML-Noise more optimally than Song, while auditory neurons in control birds encode both ML-Noise and Song equally well. Even upon adjusting for firing rate differences using a general linear model, wn-reared neurons have higher Gamma Information values than the control neurons for both Song and ML-Noise, with a larger effect for ML-Noise (see Results). B. To control for potentially small differences in the types of receptive fields found in wn-reared birds compared to control birds, we also estimated the Gamma Information (bits/s) for single neurons in control birds that had similar (‘matched’) SIs to the receptive fields found in wn-reared birds. The pairwise difference in Gamma Information for Song vs. ML-Noise is still negligible in the matched control case, while the neurons in wn-reared birds encode for ML-Noise more optimally than Song. Even upon adjusting for firing rate differences using a general linear model, neurons from the wn-reared condition still have higher Gamma Information values than the matched control neurons for both Song and ML-Noise, and once again the effect size is larger for the ML-Noise stimuli than for the Song stimuli (see Results).

In line with our previous work [28], the Gamma Information values obtained for song and ML-Noise in field L neurons of control birds were approximately identical (pairwise difference for control = −0.2 bits/s, t(112) = 0.71, p = 0.480; and pairwise difference for ‘matched’ control neurons = −1.8 bits/s, t(26) = 1.43, p = 0.168) (Figure 6). In that work, we had also reported higher Gamma Information rates for ML-Noise at lower levels of the auditory system (i.e. in the avian inferior colliculus or Mesencephalicus Lateralis pars dorsalis (MLd)), but the effect was reversed at higher levels of the auditory system (i.e. in the Caudo Lateral Mesopallium or CLM). We interpreted those results as an increase in selectivity as sound traverses the auditory processing stream for sound features that are important for distinguishing among songs. The higher information rates for noise-like sounds found at lower levels can be explained by the higher entropy of ML-Noise stimuli over song stimuli: ML-Noise stimuli are more different from each other than song stimuli.

Consistent with the higher values from the STRF predictions for ML-Noise in this subset of wn-reared neurons, we found an enhanced representation for ML-Noise in these wn-reared neurons in the MI calculations as well (Figure 6). First, we found an increase in pairwise Gamma Information rates for ML-Noise compared to song in the wn-reared animals that was not present in the control or matched control group: pairwise difference in Gamma Info for Con (song) versus ML-Noise = −5.2 bits/s, t = −2.35, p = 0.026 (Figure 6). Additional non-parametric tests confirmed the statistical robustness of this effect. In control birds, a greater number of neurons preferred song, although this effect was not significant (# of cells preferred song = 61 vs # of cells preferred ML-Noise = 52, Sign test: p = 0.451). In contrast, in the wn-reared birds, we found the opposite sign for the effect (# of cells preferred song = 6, # of cells preferred ML-Noise = 21, Sign test: p = 0.005). The ranksum test also showed a significant difference between the two rearing conditions (p = 0.003).

Second, we compared the Gamma Information across rearing conditions. Since MI depends on a neuron's firing rate and its z-score (e.g. [28]), we used a general linear model analysis in which z-scores and rearing conditions were used as predictor variables to estimate information rates. As expected, this analysis reproduced the significant effect of z-scores: Gamma Information rates increased as z-scores increased in both rearing conditions (data not shown). After taking this correlation into consideration, we found the subset of wn-reared neurons had higher information than the control neurons for both song (Gamma Info difference = 5.2 bits/s, F(1,137) = 22.64, p<10^−4^) and ML-Noise (Gamma Info difference = 9.1 bits/s, F(1,137) = 29.37, p<10^−4^). Although both increases are highly significant, the effect size was almost twice as large for ML-Noise (ES = 0.93) than for song (ES = 0.46). Thus, even after adjusting for firing rates, this subset of wn-reared neurons had higher Gamma Information rates than the control neurons but this increase was larger for ML-Noise stimuli. We repeated this general linear model across-comparison analysis with the matched control group. A similar effect was observed: neurons from the wn-reared condition had higher information values than the matched control neurons for both song (Gamma Info difference = 5.11 bits/s, F(1,51) = 7, p = 0.01) and ML-Noise (Gamma Info difference = 8.6 bits/s, F(1,51) = 7.08, p = 0.01), and once again the effect size was larger for the ML-Noise stimuli (ES = 0.57) than for the song stimuli (ES = 0.17).

Even though we found similar or greater information rates for song in this subset of wn-reared neurons, we show below that as an ensemble this subset is still deficient in coding for song compared to control animals. Moreover, we remind the reader that in general most wn-reared neurons had depressed responses to song and greater variability, which renders the calculation of their Gamma Information rates unreliable and with small expected values. An even more interesting finding is that the single neuron information rates for ML-Noise stimuli increased in wn-reared animals. These single neuron information findings lay the foundation for interpreting the ensemble information analysis described below.

#### Reduced ensemble encoding and greater redundancy for song

**Figure 7:**
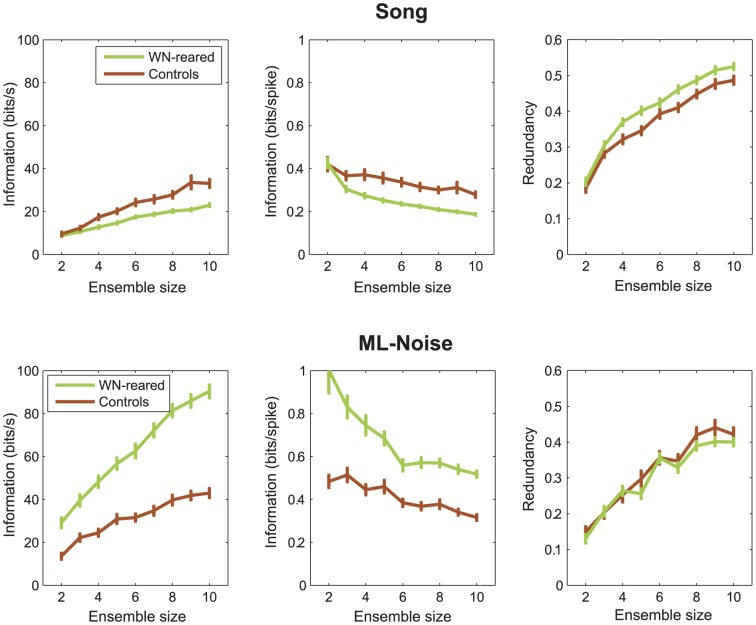
Ensemble neural coding for song more redundant in wn-reared birds. A. Mutual Information for neuronal ensembles of two to ten neurons for the control and the wn-reared cases in response to Song (on the left panel, in bits/s and on the middle panel, in bits/spike). Error bars represent one standard error obtained by randomly sampling neurons from our dataset. Redundancy in information transmitted as a function of the number of neurons for the control and the wn-reared cases in response to Song (on the right panel). Errors bars represent one standard error obtained by randomly sampling neurons from our dataset. B. Same as in **A** but for ML-Noise stimuli.

Next, we investigated how ensembles of neurons convey information about song versus ML-Noise, with a particular focus on examining the redundancy of the neural code. Since wn-reared birds were never exposed to song of either conspecifics or fully-audible versions of autogenous song, we hypothesized that this ensemble of wn-reared neurons (n = 27) might not have been subject to the level of stimulus-driven neural competitive mechanisms that might result in an efficient representation of song, characterized by low redundancy. To estimate the redundancy, we calculated the ensemble mutual information (MI) and compared it to the sum of the MI for each neuron in the ensemble (see Methods). As expected, increasing the ensemble size of neurons increased the MI rate (bits/s) in response to both song (top left panel, Figure 7) and to ML-Noise (bottom left panel, Figure 7). Additionally, and in accordance with the single neuron analysis, the ensemble MI rate in response to ML-Noise in wn-reared birds was greater than in control birds (bottom left panel, Figure 7), whereas this effect was reversed upon increasing the ensemble size (n>3) in response to song (top left panel, Figure 7). Similarly, when firing rates were taken into account to obtain measures of information per spike, increasing the ensemble size (n>2) further decreased the MI per spike for song in wn-reared birds compared to control birds (top middle panel). In other words, this subset of wn-reared neurons conveyed information about song less efficiently than the ensemble of neurons in control birds. The reverse was true for the encoding of ML-Noise (bottom middle panel), in that the neural spikes obtained from wn-reared birds are extremely discerning of ML-Noise, as also evidenced in the single neuron analysis above. The redundancy analysis (right panels, Figure 7) confirmed these effects. As the number of neurons in the ensemble increases (n>3), ensemble responses from wn-reared birds showed a greater increase in coding redundancy for song than ensemble responses from control birds (top right panel), whereas there was no difference in redundancy for ML-Noise (bottom right panel). In other words, experience with song seems crucial for developing neural representations for distinct complex features in song that would result in an efficient and non-redundant neural code.

## Discussion

In this study, we examined the effects of early noise exposure and social isolation on the processing of simple and complex sounds, including communication signals, in the avian primary auditory cortex analog (the field L complex) of adult zebra finches. We provide the first evidence that the absence of patterned auditory stimulation during postnatal life did not play a role in establishing neural tuning to simple frequency-based sounds in all the auditory cortical laminae, but dramatically reduced the neural responsivity and selectivity for natural sounds, such as song, over some synthetic sounds in the superficial and deep laminae of field L, and, for the subset of field L neurons that responded faithfully to song (mostly from the thalamorecipient lamina), a significant increase in the redundancy of the ensemble neural code. We thus conclude that layer-specific differential development of the auditory cortex and the specialized auditory cortical responses to particular higher order sound patterns found in vocalizations required exposure to these complex sound patterns and/or social experience, novel findings that otherwise could have easily been missed had we probed the system with only simple synthetic stimuli.

Previous research in mammalian systems has shown that postnatal exposure to white noise leads to immature auditory cortical map formation (i.e. an overrepresentation of high frequencies in the simple tonotopic maps) with a prolonging of the critical period in young rodents [13], [41], [42], [43] and a return to critical period-like plasticity in adult rodents [17]. Developmental arrest could also be localized to specific regions of the auditory cortex by exposure to band-limited noise [44]. Substantial structural and functional concordance between the layers of A1 and subfields of L, as evidenced by similarities in cell morphology, intratelencephalic connections, gene expression, functions, and radial-columnar architecture [45], [46], [40], might suggest comparable outcomes for A1 and L under similar environmental conditions (i.e. noise-rearing). However, the results from our noise-reared songbirds could not simply be explained by a uniform developmental delay in functional maturation. First, in contrast to the maintenance of a juvenile tonotopic map representation in layers IV-V of noise-reared adult A1 [13], [17], we report no developmental arrest for frequency-intensity tuning as assessed by the overall normal responses to tone-based simple synthetic sounds, such as Pips and Tones, in field L of noise-reared birds (Figure 4A). Indeed, unlike juvenile field L neurons that display an overall limited/immature neural responsivity for Pips and Tones [27], our noise-reared field L neurons were developmentally mature in their response strength to these simple synthetic sounds.

Furthermore, our overall responsivity and song-selectivity measures in the thalamorecipient L2 sub-region (homologous to layer IV in A1) were similar in wn-reared and control birds (Figure 5), which is in sharp contrast to the developmental disruption mapped in the middle layers (IV-V) of A1. L2 was the only sub-region that developed overall song-selectivity independent of patterned input, whereas superficial and deep laminae L1 and L3 required patterned spectro-temporal input for the emergence of their overall song-selective properties. This neurodevelopmental process of the lower-level thalamo-recipient lamina developing before downstream or more specialized laminae is shared by other primary sensory cortices (V1: [47]; S1: [48]) and has yet to be shown in studies of A1.

A third feature, distinct from noise-exposed rodent studies, is comparable STRFs obtained from controls and those obtained from a subset of phase-locked and stimulus-driven wn-reared neurons (mostly L2 neurons). Our results, albeit based on a small dataset, showed no differences in proportions of narrowband, broadband, wideband, offset, hybrid, and complex neurons, even though the adult STRFs were derived from all subfields of L. Furthermore, we did not find any differences in the bandwidths of our noise-reared and control STRFs, whereas [13] and [17] report larger bandwidths for frequency tuning in noise-reared animals (and similar to juveniles) compared to normal adults. However, it is possible that our STRF estimation method constrains the quality of data we use moreso than previous reports in A1. Another caveat is that we do not know whether the neurons for which we could not obtain STRFs had changes in frequency tuning bandwidth, which could have been assessed from intensity threshold measures. Nevertheless, as discussed below, we did find a number of very important differences in neural response properties between our control and wn-reared animals.

Although responses to simple tones in all regions and receptive field properties in L2 remained normal, social and acoustic isolation from patterned sounds resulted in a profound reduction in the overall field L neural response strength and selectivity for complex and natural sounds, such as song: overall neural responses to more complex sounds, such as Ripples, WN and Con, did not mature to the levels found in control adults (Figure 4a), and neural selectivity for Con over Pips and Con over Ripples was less than half of control zebra finches, while selectivity for Con over Tones was completely missing (Figure 4b). The overall firing-rate based tuning to Tones (a broadband stimulus set with an identical overall power spectrum as song but lacking specific spectro-temporal structure such as harmonic stacks or tempo of song) reveals that as opposed to controls and also juveniles, wn-reared neurons were preferentially tuned to sound features that were simpler than those found in complex communication signals. We also report that reduced song-selectivity was a characteristic of wn-reared subfields L1 and L3 (Figure 5b), a finding consistent with studies in other sensory systems, where neurons in extrathalamic layers show greater plasticity and have a longer experience-dependent critical period than do layer IV neurons [47], [48].

Taken together, our results provide additional evidence for the neurodevelopmental principle that the initial establishment of topographic mapping (or simple tone-based responses in our case) within developing cortical circuits is shaped by innate mechanisms and is primarily independent of experience (and first demonstrated by the vision-independent emergence of orientation-maps and ocular dominance columns in V1 [49], [50], [51]). However, sensory experience is essential for the development of specific features of these maps, as in the optimal neural representation for noise-like sounds in our wn-reared birds or reduced redundancy for song in the case of our control birds (discussed below).

We also report that exposure to patterned spectro-temporal sounds and higher order sound features is required to increase neural spiking reliability in subfields L1 and L3 (Figure 5c), as evidenced by relatively smaller FF measures for control birds. Increased neural variability was an additional factor that deterred reliable STRF estimation for most wn-reared neurons, excepting a subset that reliably phase-locked to complex stimuli (and mostly from L2). Sluggish temporal responses were a prominent feature of noise-rearing in A1 as well [16], and explained by changes in cellular microcircuitry physiology [52], [42], [43] towards reduced cortical inhibition, similar to juvenile A1 [17].

Finally, our analysis revealed a significant change in coding properties yet to be examined in mammalian systems: we found that for the subset of neurons for which we obtained STRFs, white noise exposure during development led to a more efficient representation of noise-like sounds, such that single wn-reared neurons encoded ML-Noise more efficiently (Figure 6) than they did song, a pattern not observed in control birds. Because ML-Noise stimuli had higher entropy than song stimuli, one would expect *a priori* higher MI values for ML-Noise than song [28]. However, the higher Gamma Information values in the wn-reared animals were not simply a reflection of differences in stimulus entropy since experience with natural sounds resulted in the elimination of this effect, as comparable Gamma Information rates for both song and ML-Noise were found in control animals (Figure 6). A more likely explanation is a “positive” effect of rearing (possibly due to experience-dependent synaptic mechanisms) towards a more efficient representation for noise-like sounds in wn-reared birds and/or a less redundant representation for song in control birds.

We also examined the effect of wn-rearing on the ensemble coding properties of this subset of neurons. We had previously shown that single neurons in field L (and higher auditory areas) in socially-raised adult birds efficiently represented song features as reflected by higher information rates [28]. In this work, we demonstrated lower redundancy of the neural code in control birds (Figure 7), supporting a more efficient representation for song features at the ensemble level. We then provided evidence that this less redundant representation develops at least partially as a result of normal experience with patterned acoustic stimuli: we found a striking and very significant increase in the redundancy of the ensemble code for song stimuli (but not ML-Noise) in wn-reared animals. We do not know, however, whether the observed changes are permanent, which would imply a critical developmental period for this optimization process. Further studies are needed to assess the potential of restoring normal auditory responses to complex natural sounds in adult animals raised in noisy environments and subsequently exposed to normal acoustical stimulation.

On a mechanistic level, it has been suggested that a sparse (and thereby less redundant) representation of behaviorally-relevant stimuli could be achieved by local Hebbian mechanisms (neurons that learn higher-order common patterns, wire together) and by the development of synchronous excitatory and inhibitory networks of neurons that use spike timing-dependent plasticity (STDP) mechanisms [53], [54], [55], [56], [57]. Repeated exposure to behaviorally-relevant stimuli could then result in the development of coordinated excitatory and inhibitory neural ensembles selective for higher-order stimulus correlations and sparse representation of such stimulus structure, creating a non-redundant network. Since spike timing-dependent and rate-dependent LTP/LTD mechanisms have been shown to shape song-selective neurons in song nuclei [58], [59], and tightly coupled excitatory and inhibitory neurons are known to play a key role in processing auditory information in songbirds [60], [61], it is possible (and remains to be tested) that patterned acoustic stimuli engage similar experience-dependent mechanisms in the songbird auditory networks for the overall development of temporal precision and joint spectro-temporal tuning in the majority of field L neurons and the emergence of specialized and non-redundant ensemble coding for song.

From a functional point of view, it has been proposed that ‘sparse coding’ is important for efficiently representing complex stimuli because higher-level stimulus structure is represented in terms of lower-level response statistics [62]. For audition in songbirds, a sparse representation of song features could then be used in networks tuned for even higher-level tasks such as the recognition of a particular song or the memorization of a tutor song [63]. The same concept has also been applied to explain the role of sparse representations found at high-level nuclei in several sensory modalities and animal model systems [64], [65], [2], [66], [67], [68], [69], [70]. One of the key aspects of the sparse coding theory is that the optimal neural representation is tightly linked to the statistics of the stimuli: for example, the set of V1 receptive fields are derived from natural images [64]. However, the causal link in this relationship had never been proven. Here we show for the first time that indeed when the statistics of the stimuli were changed during rearing, the redundancy in the neural representation for natural sounds increased or, equivalently, that the ensemble sparseness significantly decreased. This increase in redundancy for song was also correlated with an overall loss of communication signal-selective responses that we report here for other field L sub-regions.

In summary, our study provides insights on the interaction between innate and experiential factors in the development of complex response properties of the auditory cortex. Patterned acoustic input appears to play no role in the initial establishment of neural tuning to simple frequency-based sounds in all layers, or the spectro-temporal receptive fields readily sampled in L2/Layer IV, lending support to the idea that an innate and relatively stable topography serves as a form of cortical representational stability. However, patterned input, such as rich acoustic and social experiences, is required for the next stage of network developmental changes, in which responses in superficial and deep layers become more temporally precise and selective for complex acoustic ‘objects’, and all layers create more complex and efficient neural representations of native sounds. Although optimization of sensory systems likely occurs on evolutionary, developmental, and behavioral timescales, our results underscore the significance of the developmental timescale in optimizing the auditory system for complex natural sounds, such as vocalizations. Our findings also imply that socially-impoverished or noisy environments could adversely affect essential perceptual abilities and cause developmental delays in both songbirds and humans, and further investigations are required to examine the potential to reverse such developmental delays.
